# A cohort study evaluating the association between concurrent mental disorders, mortality, morbidity, and continuous treatment retention for patients in opioid agonist treatment (OAT) across Ontario, Canada, using administrative health data

**DOI:** 10.1186/s12954-020-00396-x

**Published:** 2020-07-23

**Authors:** Kristen A. Morin, Joseph K. Eibl, Graham Gauthier, Brian Rush, Christopher Mushquash, Nancy E. Lightfoot, David C. Marsh

**Affiliations:** 1 Northern Ontario, School of Medicine, Sudbury, ON Canada; 2 Health Sciences North Research Institute, Sudbury, ON Canada; 3 Centre for Addiction and Mental Health, Toronto, ON Canada; 4 Lakehead University, Department of Psychology, Thunder Bay, ON Canada; 5 Laurentian University, Sudbury, ON Canada; 6 ICES, Toronto, ON Canada

**Keywords:** Opioid use disorder, Opioid agonist treatment, Mental disorders

## Abstract

**Background:**

Due to the high prevalence of mental disorders among people with opioid use disorder, the objective of this study was to determine the association between concurrent mental disorders, mortality, morbidity, and continuous treatment retention for patients in opioid agonist treatment in Ontario, Canada.

**Methods:**

We conducted a retrospective cohort study of patients enrolled in opioid agonist treatment between January 1, 2011, and December 31, 2015. Patients were stratified into two groups: those diagnosed with concurrent mental disorders and opioid use disorder and those with opioid use disorder only, using data from the Ontario Health Insurance Plan Database, Ontario Drug Benefit Plan Database. The primary outcome studied was all-cause mortality using data from the Registered Persons Database. Emergency department visits from the National Ambulatory Care Database, hospitalizations Discharge Abstract Database, and continuous retention in treatment, defined as 1 year of uninterrupted opioid agonist treatment using data from the Ontario Drug Benefit Plan Database were measured as secondary outcomes. Encrypted patient identifiers were used to link information across databases.

**Results:**

We identified 55,924 individuals enrolled in opioid agonist treatment, and 87% had a concurrent mental disorder diagnosis during this period. We observed that having a mental disorder was associated with an increased likelihood of all-cause mortality (odds ratio (OR) 1.4; 95% confidence interval (CI) 1.2–1.5). For patients diagnosed with mental disorders, the estimated rate of ED visits per year was 2.25 times higher and estimated rate of hospitalization per year was 1.67 times higher than for patients with no mental disorders. However, there was no association between having a diagnosis of a mental disorder and 1-year treatment retention in OAT-adjusted hazard ratio (HR) = 1.0; 95% CI 0.9 to 1.1.

**Conclusion:**

Our findings highlight the consequences of the high prevalence of mental disorders for individuals with opioid use disorder in Ontario, Canada.

## Introduction

The expanding opioid crisis in Canada is a complex issue that is exacerbated by factors such as increased exposure to prescription opioids [1], the prevalence of untreated mental illness [2], social isolation [3], a health system that does not work across silos, and largely unregulated advertising practices by drug companies [4, 5].

Research examining substance use and psychiatric comorbidities reports that approximately 50% of people with opioid use disorder (OUD) receiving treatment have a lifetime psychiatric diagnosis [6–10]. It has been shown that people who use opioids and psychiatric comorbidities have high all-cause mortality [7]. In comparison to southern and urban communities, communities in northern Ontario [11–15], Canada, including First Nations, rural, and remote communities, generally experience high poverty, have access to limited infrastructure and health resources, demonstrate high risk-taking behaviors, and have less control over their environment specific to weather and occupation. Moreover, in Ontario, mental health services are seldom available or even coordinated with OUD services. Such factors pose an increased risk for mental disorders, substance use, and suicide [16]. Given the confluence of emergent factors and the ongoing nature of the relationships between these factors, it is likely that challenges with opioids will continue in Canada.

Opioid agonist treatment (OAT) is currently the intervention with the best evidence for long-term treatment of OUD [17]. However, retention in treatment continues to be a barrier to successful clinical outcomes for individuals with OUD. It is common for OAT patients to cycle through treatment and re-initiation of opioid consumption before they are stabilized in care, which can be dangerous because of changes to opioid tolerance in patients leading to a higher risk of mortality [18–23]. Therefore, despite the availability of evidence-based treatment for OUD, opioid-related deaths continue to be a critical issue in Ontario [24], the most populated province in Canada. For instance, from 2016 and 2018, 9078 Ontarians died of opioid poisoning [25]. There has been a surge in opioid-related hospitalizations and emergency department (ED) visits [26–28], increasing from 9.42 per 100,000 population in 2003 to 19.55 per 100,000 population in 2015 [29].

According to the literature, mental disorders are prevalent among people with OUD [6, 7, 30]. Also, there are problems with the existing literature, including small sample sizes, restricted jurisdictions, and lack of longitudinal studies, which is an issue because OUD and mental disorders are chronic relapsing conditions [31, 32]. Also, previous studies have mainly focused on methadone as a form of OAT. However, buprenorphine prescriptions for the treatment of OUD are increasing, especially in rural areas of Ontario [33]. Lastly, population-level issues such as mortality, ED visits, and hospitalizations [27, 34] and their relation to mental disorders have not been adequately researched.

The goal of this study was to highlight the effects of the chronic nature of opioid use disorder (OUD) and mental disorders on population health and address some of the gaps in the literature related to OUD and mental disorders. We evaluated the associations between concurrent mental disorders and mortality, morbidity, and continuous treatment retention in patients within OAT across Ontario. Our secondary objective was to examine regional variation in health service usage and treatment retention because health care services are materialized differently across Ontario, thus potentially resulting in varying OAT outcomes [27, 35, 36]. We hypothesized that OAT patients with mental disorders had poorer outcomes compared to OAT patients with no mental disorders.

## Methods

### Study design

We conducted a retrospective cohort study between January 1, 2011, and December 31, 2015, in Ontario. All health services examined in this study (except prescription drug) are covered by a government single-payer insurance program called the Ontario Health Insurance Plan (OHIP). Therefore, the study included all patients in Ontario who had accessed OAT within the study period. We used the date of the first opioid visit as the index date for all analyses. We designed the study around the index date, and follow-up times were fixed to eliminate bias related to the varying length of time in treatment for each patient. The last date of inclusion was December 31, 2015, and all dependent and independent variables were evaluated for 1 year after enrollment in OAT.

A treatment episode was defined as the time between the last and first OAT billing codes within a period of continuous retention in treatment (no interruptions care of more than 30 days). We used the first episode of OAT to identify patients, meaning that there was no previous history of OAT (including methadone or buprenorphine/naloxone) in the year before the first treatment episode. We chose to only include patients with no history of OAT in the year before the first episode during the study period to eliminate bias associated with cases involving multiple treatment attempts [23, 37, 38].

This study was approved by the Research Ethics Board of Laurentian University in Sudbury. This study is reported following the Strengthening the Reporting of Observational Studies in Epidemiology (STROBE) guidelines [39]

### Study population

The study cohort was created by extracting OAT patients from the Ontario Drug Benefit Plan (ODB) database, which captures data on publicly funded pharmacy billing using drug identification numbers (DIN) (see Additional file 1) and with the (OHIP) database, which captures data on publicly funded physician-based health services using physician billing codes (see Additional file 2). In previous published ICES studies [27, 35, 40], the ODB database was used as the primary source to identify OAT patients. However, in Ontario, residents are only eligible for ODB public drug coverage if they are aged 65 years or older, reside in a long-term care facility, are disabled, are receiving social benefits for income support, or have high prescription drug costs relative to their net household income. Since 2011, new billing codes have helped to identify OAT services [41] in administrative databases because OHIP coverage is available to all permanent residents of Ontario. Therefore, to avoid excluding a subset of the population and risking selection bias, we used both ODB and OHIP databases to identify the primary patient cohort.

**Fig. 1:**
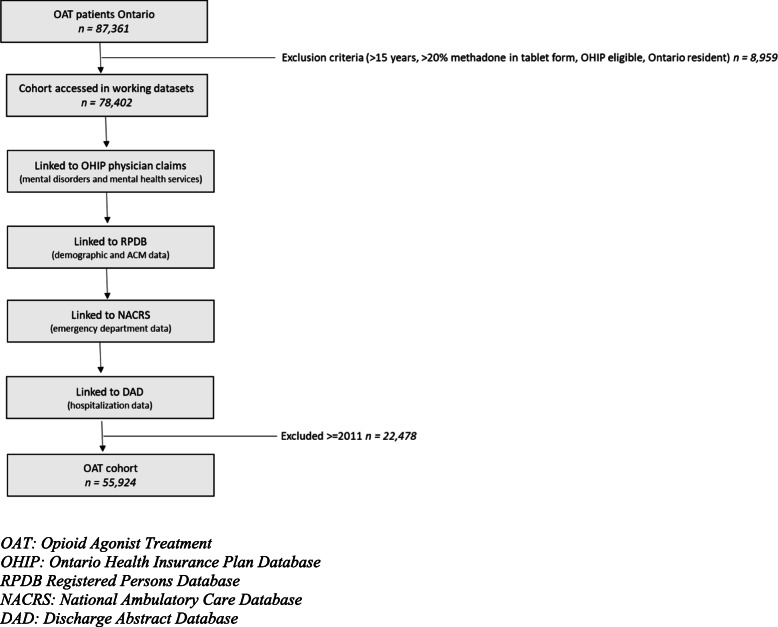
Flow chart outlining data build including linkages*.* OAT, opioid agonist treatment; OHIP, Ontario Health Insurance Plan Database; RPDB, Registered Persons Database; NACRS, National Ambulatory Care Database; DAD, Discharge Abstract Database

A set of exclusion criteria was applied to define the main cohort. We excluded all patients under 15 years of age (*n* = 2535 patients). Patients who were identified in the ODB with over 20% of their methadone dose in tablet formulation over 1 year were excluded because methadone in the tablet formulation is not approved for use as an addiction treatment in Canada (*n* = 5560 patients). Additionally, the following patients were excluded from the study: patients who were not eligible for OHIP (*n* = 437 patients) and non-Ontario residents (*n* = 427 patients). Ontario residents may not be eligible for OHIP coverage if they have been absent for more than 30 days during the first 6 months of residence in the province and if they were not in Ontario for at least 153 days in 12 months. Patients identified from ODB (*n* = 1383 patients), from OHIP (*n* = 30,124 patients), and patients who were identified in both databases (*n* = 24,417 patients) were combined to create the primary cohort (*n* = 55,924 patients). The steps used to create the primary cohort are outlined in Fig. 1.

### Data sources

We obtained anonymized patient-level data from Ontario publicly funded health services by submitting a formal requisition to ICES. ICES, previously known as the Institute for clinical evaluative sciences, is an independent research institute that collects and analyzes health care data for research. Patient information was linked anonymously across databases using encrypted ten-digit health card numbers. The linking protocol is used routinely for health system research in Ontario [42–44].

All diagnostic information from physician visits was determined using billing data from OHIP. ED visits were identified using the National Ambulatory Care Reporting System (NACRS). Hospital admissions were identified using the Discharge Abstract Database (DAD). We obtained patients’ location of residence and demographic information, including all-cause mortality from the Ontario Registered Persons Database (RPDB), which contains unique data for each resident who has ever received insured health.

### Patients with a diagnosis of one or more mental disorders

Exposure to one or more mental disorders was assumed for all patients who had at least one of the OHIP diagnoses listed in Additional file 4. We excluded any substance-related diagnoses from our mental disorder diagnosis definition, which means that all patients in the mental disorders group had OUD and one or more mental disorders other than substance use disorders. We used OHIP diagnosis codes to identify patients in the mental disorders group. In the database, there is one general substance use diagnosis code. All substance use disorders that fall under code 304 are listed in Additional file 4. All patients in the cohort had that code based on their opioid dependence. Therefore, there was no way to detect those who also had a dependence on other substances such as cocaine, benzodiazepines, or other substances.

Patients were assigned to only one of the following groups: those diagnosed with a mental disorder other than OUD and those not diagnosed with a mental disorder other than OUD. The total time parameter to identify the diagnoses of mental disorders was 2 years for each patient. We identified patients who had a mental disorder diagnosis 1 year before the index date to 1 year after the index date (or the study end date). Mental disorders can be chronic, re-occurring conditions; therefore, the broad time frame was chosen to capture the condition accurately. Such wide time parameters are commonly used in other studies examining mental health comorbidities using administrative databases [45–49].

### Outcomes

Study outcomes were defined based on the need to assess the association of all-cause mortality, frequent ED visits, and hospitalizations as a function of concurrent mental disorders and OUD, and not as an exposure leading to an event. All-cause mortality, ED visits, and hospitalizations have been used as indicators of complexity in the OUD population in other studies [7, 27, 50, 51]. Additionally, both frequent ED visits and hospitalizations are metrics used by health system planners and funders in Ontario to understand gaps in services in communities [50–52].

We requested the all-cause mortality variable from ICES as a dichotomized variable. At the time of the study, mortality-specific data were not available for the entirety of our study period. We used data from the RPDB database to calculate the number of days to death date from the study index date for each patient in the cohort to create the variable. If the patient had a mortality event anytime between their index date and the end of the study period (December 31, 2016), we assigned a code of 1 (all-cause mortality) or 0 (no all-cause mortality).

We used data from the NACRS database to identify ED visits. We considered a patient as having frequent ED visits if (1) contact with ED was after the index date and (2) a patient had ten or more ED events in a publicly funded Ontario hospital within 1 year. We used the diagnostic code that accompanied each ED event in the NACRS to categorize types of ED visits. Opioid-related, mental health-related, and reasons other than mental health or opioids were included in the analysis. Cutoffs for dichotomization were based on the frequency and distribution of ED visits for the entire cohort. The median number of ED visits was 14 per year, the mode was 6, and the mean was 22 (SD = 30.9). We chose to dichotomize with a value between the mean and mode (code 1 for ten or more ED visits and 0 for less than ten visits per year).

We used the DAD database to identify hospitalization. Hospitalizations were captured in three groups: opioid-related, mental health-related, and for reasons other than mental health or opioids using the primary diagnosis code that accompanied the hospitalization event in the DAD database. Hospitalizations were dichotomized and counted if a hospitalization discharge record appeared after a patient’s index date in a publicly funded Ontario hospital. The cutoff of one hospitalization was decided based on the frequency distribution of the number of hospitalizations for the cohort. The mean number of hospitalization per year was 3.

We conducted a subgroup analysis of 1-year treatment retention using the ODB database (*n* = 25,800). One-year treatment retention is correlated with a variety of positive health outcomes for patients, including reduced rates of drug use, criminal activity, and an increase in employment [53]. One-year retention in OAT was assessed based on doses dispensed (from the ODB database). If the difference between the last and first days of dispensed medication within a period of continuous retention in treatment (no interruptions in prescribed doses > 30 days) was greater than 365 days, then the patient was considered to be retained for 1 year in OAT. Thirty days was chosen based on the use of this interval in previously published research [18, 53, 54]. The database used for medication dispensing in this study might not capture doses administered in a hospital or provincial correctional settings. However, in Ontario, patients will typically continue to receive methadone or buprenorphine in these settings. Since most hospital admissions or provincial incarcerations are less than 30 days, this approach allowed us to conduct the analysis without misinterpreting such events as treatment interruption.

### Baseline covariates

The covariates available for the study included age; sex; location of residence; income quintile; human immunodeficiency virus infection (HIV); and deep tissue infection including endocarditis (OHIP diagnostic code 429), osteomyelitis (OHIP diagnostic code 730), and septic arthritis (OHIP diagnostic code 711).

### Statistical analysis

Descriptive statistics summarize the characteristics of both the patient groups. We used standardized differences to compare categorical variables and the Wilcoxon rank-sum test to compare continuous variables between exposure groups. Standardized differences are not dependent on sample size; therefore, it was an appropriate measure to summarize characteristics of the large sample of patients in this study [55]. Most researchers suggest that a value of standardized differences (d) ≥ 10% indicates a balance between groups [56]. The group of patients not diagnosed with mental disorders was considered the baseline comparison. Covariates included age, sex, location of residence, income quintile, HIV status, and deep tissue infection.

A logistic regression model was used to test the association between mental disorders and all-cause mortality. We adjusted the model using the covariates and calculated unadjusted and adjusted odds ratios (OR) and 95% confidence intervals (CI). If the OR was equal to one, having a mental disorder did not affect the likelihood of all-cause mortality. If the OR was more than one, having a mental disorder was associated with the likelihood of all-cause mortality. If the OR was less than one, having a mental disorder was associated with a decreased likelihood of all-cause mortality. If the CI spanned one, it was evidence of a lack of association between the exposure and the outcome [57].

Negative binomial regression models were used to estimate the association between mental disorders, ED visits, and hospitalizations. In a study comparing different models to evaluate health service utilization, the negative binomial regression model was found to be one of the most appropriate because it can address over dispersion that is typically observed in health care utilization data [58]. We used the regression coefficient to calculate OR and 95% CI [58, 59].

A Cox Proportional Hazard Model was used to characterize the risk of treatment discontinuation over time for both methadone and buprenorphine/naloxone for the subgroup of patients identified through the ODB database (*n* = 28,000). Due to the time frame of the study, we assumed a constant hazard ratio between groups over time. We adjusted the model using the baseline covariates and calculated hazard ratio (HR) and 95% CI. If the HR was equal to one, having a mental disorder did not affect the likelihood of 1-year treatment retention. If the OR was more than one, having a mental disorder was associated with the likelihood of 1-year treatment retention. If the OR was less than one, having a mental disorder was associated with a decreased likelihood of 1-year treatment retention. If the CI spanned one, it was evidence of a lack of association between the exposure and the outcome. All statistical analyses were conducted from the secure server using SAS Version 9.4 [60]. Data was reviewed by ICES to insure privacy standards were met.

## Results

A total of 55,924 patients who were enrolled in OAT between 2011 and 2015 were identified. Of those, 48,679 (87.0%) had one or more diagnoses of mental disorder. We followed all patients for 1 year, and there were 2712 (8.2%) deceased patients during follow-up. There were 534 records with missing information on income quintile and 3 records with missing information on the location of residence. Missing income records were re-assigned to the lowest income group, and those with missing information on the location of residence were deleted.

### Prevalence of mental disorders

**Fig. 2:**
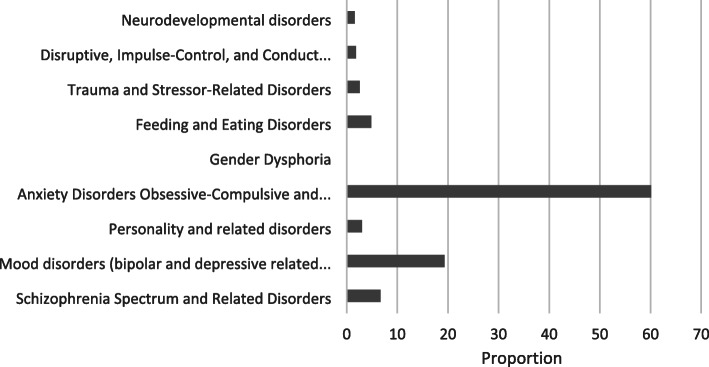
Proportion of diagnoses of mental disorders for patients enrolled in OAT

Anxiety disorders, including obsessive compulsive disorder and other anxiety-related disorders (60%) and mood disorders, including bipolar and other depressive-related disorders (20%), were among the most prevalent in the cohort. The results are presented in Fig. 2.

### Patient characteristics

Males represented a lower proportion of individuals in the mental disorders group (63.0%) compared to the group with no mental health disorder (77.0%, (*p* < 0.001)). The proportion of individuals aged 15–24 in the mental disorders and no mental disorders groups was 17.9% and 20.9%, respectively. Of the individuals with mental disorders, 77.8% lived in southern urban regions, 4.6% in northern rural areas, 9.3% in northern urban areas, and 8.2% in southern rural areas. This compared to individuals who had not been diagnosed with a mental disorder, 71.9% lived in southern urban areas, 21.0% lived in northern rural areas, 10.5% in northern urban areas, and 9.4% in southern rural areas (sd 0.2). We observed significant differences between the two groups with regards to the proportion of patients with HIV (mental disorders group = 0.8% and no mental disorders group = 0.3%, sd 0.1) and deep-tissue infections (mental disorders group = 3.3% and no mental disorders group = 1.3%, *p* 0.1). Retention in buprenorphine treatment was significantly different between groups (mean days engaged in buprenorphine treatment was 55.0, standard deviation (SD) = 167.8, and 34.8, SD = 121.8). However, there was no statistically significant difference identified in the mean days retained in methadone treatment: 356.8, SD = 441.0 and 344.6, SD = 409.8 for the mental disorders group and no mental disorders group respectively on a subgroup of patients identified from the ODB database (*n* = 28,500). The patient characteristics are outlined in Table 1. Importantly, there was a significant difference in the proportion of patients with an all-cause mortality event between groups (mental disorders group = 5.1% and no mental disorders group = 3.1%, sd 0.1) and significant differences in the proportion of patients retained for 1 year in OAT (mental disorders group = 37.6%, and no mental disorders group = 35.7%, sd 0.2). The patient characteristics are outlined in Table 1.

**Table 1:** Patient group characteristics

Variable	Concurrent mental and opioid use disorder	Opioid use disorder only	Standardized differences (sd)
	*N* = 48,679 (87.0)	7245 (13.0)	
Age			0.3
15 to 24	8.727 (17.9)	1514 (20.9)	
25 to 34	16,148 (33.2)	2859 (39.5)	
35 to 44	10,712 (22.0)	1383 (19.1)	
45 to 54	8812 (18.1)	989 (13.7)	
55 to 64	3348 (6.9)	338 (4.7)	
+65+	932 (1.9)	162 (2.2)	
Sex			0.2
+Male	30,654 (63.0)	5575 (77.0)	
Female	18,025 (37.0)	1670 (23.1)	
Geography			0.2
+Southern urban	37,887 (77.8)	5209 (71.9)	
Northern rural	2240 (4.6)	593 (21.0)	
Northern urban	4533 (9.3)	761 (10.5)	
Southern rural	4016 (8.2)	682 (9.4)	
Income			0.0
+5	5532 (11.5)	734 (10.4)	
4	7014 (14.6)	1078 (15.2)	
3	8690 (18.1)	1282 (18.1)	
2	10,886 (22.6)	1613 (22.7)	
1 (lowest)	16,020 (33.3)	2388 (33.7)	
HIV positive	390 (0.8)	21 (0.3)	0.1
Deep-tissue infection	1584 (3.3)	92 (1.3)	0.1
All-cause mortality	2485 (5.1)	227 (3.1)	0.1
*One year continuous OAT	8757 (37.6)	903 (35.7)	0.2
*Mean days buprenorphine (SD)	55.0 (167.8)	34.8 (121.8)	< .0001
*Mean days methadone (SD)	356.8 (441.0)	344.6 (409.8)	< .0.042

*sd* standardized difference, *SD* standard deviation

*+*Reference group

*Ontario Drug Benefit plan subgroup analysis (n = 28,500)

### Outcomes

Having a diagnosis of one or more mental disorders was associated with an increase in the likelihood of all-cause mortality (adjusted OR (aOR) = 1.4; 95% confidence interval (CI) 1.2 to 1.5) when compared to patients who had not been diagnosed with mental disorders. For patients diagnosed with mental disorders, the estimated rate of ED visits per year was 2.25 times higher than for patients with no mental disorders (factor exponent (0.81) =2.25). For patients who had been diagnosed with mental disorders, the estimated rate of hospitalization per year was 1.67 times higher than for patients with no mental disorders (factor exponent (0.51) = 1.67). The results are presented in Table 2.

**Table 2:** All-cause mortality, acute care use and treatment retention in patient groups

	Patients, *N*	Outcome	Effect	95% CI
All-cause mortality		*n* (%)		
Mental disorders	48,679	2485 (5.1)	OR 1.4	1.2–1.5
+No mental disorders	7245	227 (3.1)		
ED visits		Mean (SD)		
Mental disorders	48,679	22.0 (30.9)	IRR 2.3	2.2–2.3
+No mental disorders	7245	9.8 (10.2)		
Hospitalizations		Mean (SD)		
Mental disorders	48,679	5.0 (6.8)	IRR 1.1	1.6–1.7
+No mental disorders	7245	3.0 (6.8)		
*One-year treatment retention		*n* (%)		
Mental disorders	23,268	8758 (37.6)	HR 1.0	0.9–1.1
+No mental disorders	2,532	903 (35.7)		

*SD* standard deviation, *OR* adjusted odds ratio, *IRR* incidence rate ratio, *HR* adjust hazard ratio

+ Reference group

*Ontario Drug Benefit Plan subgroup analysis (n = 28,500)

**Fig. 3:**
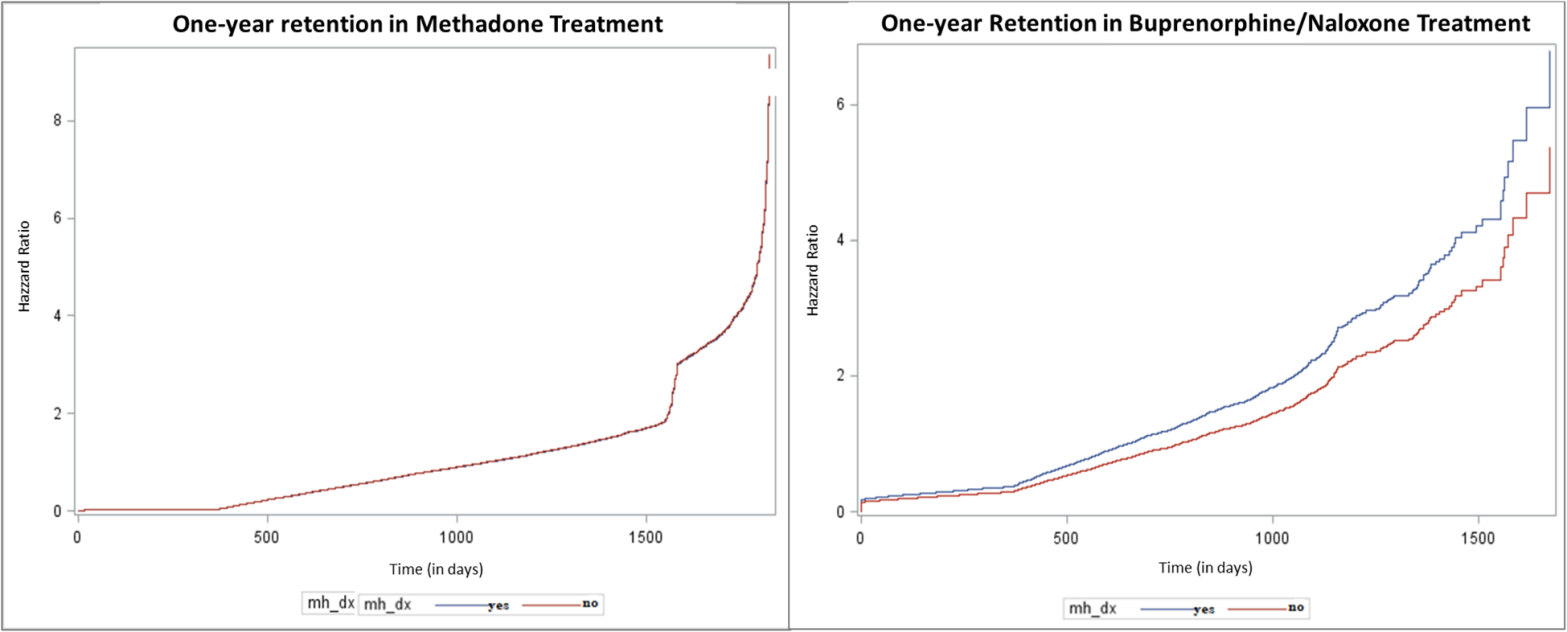
Hazzard ratio time to discontinuation for patients undergoing methadone maintenance and buprenorphine/naloxone maintenance treatment

We also found that there was no association between having a diagnosis of a mental disorder and 1-year retention in methadone maintenance treatment (adjusted hazard ratio (HR) = 1.0; 95% CI 0.9 to 1.1) in the subgroup of ODB patients. However, having a mental disorder was associated with an increased likelihood of 1-year retention in buprenorphine maintenance treatment (HR = 1.3, 95% CI 1.2–1.3) in the subgroup of ODB patients. The results are presented in Fig. 3.

### Regional differences

**Fig. 4:**
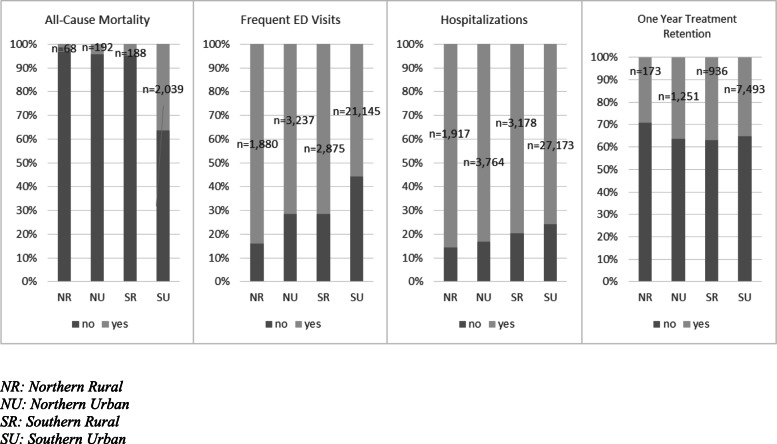
Proportion of patients for outcomes by place of residence. NR, northern rural; NU, northern urban; SR, southern rural; SU, southern urban

The highest proportion of patients who died was in southern urban areas (*n* = 2039, 5.38%) and the lowest in northern rural areas (*n* = 68, 3.04%, *p* < 0.001), whereas the highest proportion of patients with frequent ED visits was in northern rural areas (*n* = 1880, 83.93%) and the lowest in southern urban areas (*n* = 29,137, 55.81%, *p* < 0.001) (Fig. 4). Similarly, the highest proportion of patients with hospitalizations was in northern rural areas (*n* = 1917, 85.58%) and the lowest was in southern urban areas (*n* = 36,032, 71.72%, *p* < 0.001). The highest proportion of patients retained for 1 year was in southern rural areas (*n* = 347, 37.1%), followed by northern urban (*n* = 456, 36.5%), southern urban (*n* = 2630, 35.1%), and lastly northern rural (*n* = 173, 29.1%, *p* < 0.001). Results are described in Fig. 4.

## Discussion

The findings from this study indicate that patients in OAT with mental disorders have a higher likelihood of mortality, ED visits, and hospitalizations and that patients who live in northern and rural areas of Ontario have a more complex profile of health care utilization than patients who live in southern Ontario. Mortality, ED visits, and hospitalizations are important metrics used to measure health care delivery. Hospitals are acute care facilities that are not designed to treat chronic diseases such as substance use and mental disorders. Therefore, if there are high numbers of patients with OUD going to the hospital, it can be an indication that there are issues with service access in communities.

The results of this study align with the current studies which demonstrate a high prevalence of mental disorders in the OUD population [7, 30, 61]. However, in this study, 87% of the population had a mental disorder, whereas the prevalence in the literature is approximately 50% [7, 30, 61]. The high prevalence identified in our cohort may be because we examined a broad range of disorders and that our time parameter, including study time and follow-up, was much longer than in other studies [6, 7]. The study period was also much more recent and may be more representative of the current issues in Canada relating to patients in OAT. Moreover, the definition of enrollment was different from that of the previous studies because all diagnostic information from physician visits was determined using billing data from OHIP. Similar to other studies, we also found that mental disorders in the OAT population are associated with a more complex profile of health service utilization [7, 62–64].

In our study, having one or more mental disorders was associated with an increased likelihood of all-cause mortality when compared to the group that had not been diagnosed with mental disorders. This finding is consistent with a study by Saunders et al. suggesting that individuals with OUD who have psychiatric symptoms have higher rates of overdose, decreased quality of life, and higher rates of continual substance use [65]. It is important to note that the results indicate that individuals with mental health comorbidities are a complex group of patients; however, results should be interpreted with caution. It is possible that either the use of opioids is especially harmful for individuals with mental disorders or that the use of opioids might aggravate a pre-existing condition [66]. For many, OUD is a lifelong illness associated with serious chronic health and social outcomes [67]. Dichotomizing all-cause mortality allowed us to evaluate death associated with chronic mental disorders and OUD and not necessarily as concurrent disorders leading to an event. We believe that future studies should be conducted to evaluate deaths as a function of time in treatment to explore this issue further.

In addition to the high rate of mortality, we observed an association between concurrent mental disorders and high rates of ED visits and hospitalizations. This significant association is not surprising given that mental disorders have been used as a marker for greater clinical complexity by some scholars [6, 68]. The findings of this study do not show causation but associations which suggest that there may be ways to prevent serious adverse events by addressing mental health issues in concert with OUD.

Despite the differences in mortality and morbidity between the exposure groups, having one or more mental disorders was not associated with 1-year retention in methadone but an increased likelihood of retention in buprenorphine/naloxone. Some authors report that mental disorders increase retention [38, 62], and some have reported no effect [61]. It is important to note that buprenorphine/naloxone patients had lower retention rates than methadone patients (mean of approximately 40 days retained on buprenorphine vs. mean of 350 days retained in methadone) in this study and other studies in the literature [69, 70]. We believe that research exploring concepts of severity of dependence, resilience, and motivation would be critical to explain this relationship further.

We identified a trend where patients in the cohort residing in southern urban regions of Ontario had the highest prevalence of all-cause mortality. A cohort study by Gomes et al. on geographic variation in opioid prescribing and opioid-related mortality in Ontario [35] found that communities with some of the highest rates of opioid-related death were in northern Ontario. One possible factor accounting for higher mortality rates in southern urban regions is the unprecedented increased fentanyl-related deaths in recent years, which started in urban centers and is now making its way into smaller communities [71]. Another possible explanation is that barriers to access OAT are higher in northern Ontario, perhaps resulting in a larger number of deaths among individuals who were never enrolled in OAT (and thus not included in this study). It is also important to note that there may be an increased likelihood of mortality and morbidity closer to 2016 in all regions due to the increased of fentanyl in the drug supply [72].

Although the results show a lower proportion of individuals who died in northern regions, there was a significantly higher proportion of morbidity measured by ED visits and hospitalizations in rural northern Ontario. Possible factors accounting for the high prevalence of acute care use may be attributed to the well-known limited availability of specialist addiction medicine and psychiatric services in northern rural regions of Ontario [36, 73]. Lastly, with regards to geographical variation, our results demonstrated that the proportion of patients retained for 1 year of OAT treatment was highest for those living in southern rural areas. This finding is counter to the findings published by Eibl et al. [40], which demonstrated that patients living in northern rural areas were more likely to be retained for 1 year. Notably, our study is more recent than the earlier study by Eibl et al., and currently, there is a broader availability of buprenorphine/naloxone. Further research is needed on region-specific retention for individuals with concurrent mental disorders and OUD, including analysis of changes over time and the impact of changing rates of methadone and buprenorphine/naloxone prescribing.

One of the potential issues inherent to any study of this type is that health administrative data were not collected to do research, which may have led to misclassification of disease prevalence and clinical outcomes. For instance, we were not able to evaluate patients who may have a mental disorder or OUD who have not yet sought out services [74]. We did not include the Ministry of Health-funded community mental health and addiction services and federally funded health services, such as mental health and addiction services provided in First Nation communities, as well as any other mental health and addiction services funded by provincial ministries other than the Ministry of Health. Moreover, since there is a well-known lack of physicians available to diagnose and treat patients in rural areas, we may have underestimated the prevalence of mental disorders and other diagnoses.

With relation to mental diagnoses, further stratification of diagnosis type would have been of interest but was not possible as the numbers became too small to publish for the outcomes of interest. It is essential to highlight the potential underestimation of the prevalence of post-traumatic stress disorder (PTSD) since it is known to be often misdiagnosed as another anxiety, depression or other related disorders [75–78]. Details such as years of drug use, the amount or type of opioid used, the history of mental health services before 1 year before the first episode of OAT, and the number of times patients were in and out of OAT after their first episode of OAT in this study remain unknown. Additionally, although hepatitis C is highly prevalent in the OAT population and can impact outcomes, data on hepatitis C was not available for this study. In the last few years, there have been several changes concerning hepatitis C treatment availability and coverage in Ontario. Since we used physician billing data and prescription data to ascertain diagnoses in this study, we were not able to define an accurate hepatitis C diagnosis during the time of the study due to all the changes in hepatitis C treatment. Since this study is observational in nature, causality cannot be inferred. Additionally, there is a potential for associations to be significant by chance due to the large sample size.

There are also limitations associated with the way we measured the outcomes for this study. Some authors have stated that creating categories from continuous data can lead to loss of information [79]. More specifically, there are limitations to using all-cause mortality as a dichotomized variable without censoring for time. For instance, for those patients where all-cause mortality occurred during the first year of OAT, the likelihood of 1-year retention is reduced. However, the function of time is a modest bias since we observed an increase in ED visits and hospitalizations in the groups with the highest mortality. The results of this study, which were found to be statistically significant, must be interpreted critically within the context of the population of interest to determine whether the results have a clinical or health system impact.

## Conclusion

The outcomes of this study have important implications for those involved in health care planning and policy development because our data suggest that the prevalence of mental disorders in the OAT population is alarmingly high and that mental disorders are associated with severe consequences. Currently, the regulations and model of care for OAT in Ontario promotes access to services but do not incentivize efforts towards coordination with other parts of the health care system, including other addiction or mental health services. Results may be generalizable in regions where OAT programs and health care regulations are similar to those in Ontario. Further study is needed to determine the effectiveness of concurrent delivery of mental health, other substance use, and OAT services.

## Supplementary information

**Additional file 1.** Drug Identification Numbers

**Additional file 2.** Addiction Medicine Fee Codes

**Additional file 3.** Mental Health Service Fee Codes

**Additional file 4.** ICD9 and 10 Codes

**Additional file 5.** Study outcomes related to concurrent mental disorders and all-cause mortality for OAT patients (Regression Output)

## Data Availability

The datasets used during the current study are not publicly available due to privacy reasons, but aggregated data are included in this published article and its supplementary information files.

## Abbreviations

OUD: Opioid use disorder
OAT: Opioid agonist treatment
ICES: Formally known as the Institute for Clinical Evaluative Sciences
ODB: Ontario drug benefit plan
SAS: Statistical Analytics Software
OHIP: Ontario Health Insurance Plan
ED: Emergency department
CIHI: Canadian Institute for Health Information
NACRS: National Ambulatory Care Reporting System
DAD: Discharge Abstract Database
RPDB: Registered Persons Database
LHIN: Local Health Integration Network
OR: Odds ratio
aOR: Adjusted odds ratio
CI: Confidence interval
ICD: International Classification of Disease
PTSD: Post-traumatic stress disorder
HIV: Human immunodeficiency virus
ODPRN: Ontario Drug Policy Research Network
