# Supplementary material for: A cohort study evaluating the association between concurrent mental disorders, mortality, morbidity, and continuous treatment retention for patients in opioid agonist treatment (OAT) across Ontario, Canada, using administrative health data
Source: Harm Reduct J. 2020 Jul 23;17:51. doi: 10.1186/s12954-020-00396-x (PMC7376938; doi:10.1186/s12954-020-00396-x)
Supplement: Supplementary file 1 — Additional file 1. Drug Identification Numbers [file 12954_2020_396_MOESM1_ESM.docx]

Appendix A

| **Drug Identification Numbers (DIN)** |  |
| --- | --- |
| **Methadone drug identification numbers** | 02241377  02247694  02394596  02394618  09850619  09852891  09857221  09857223 |
| **Buprenorphine/naloxone drug identification numbers** | 02295695  02295709  02408090  02408104  02424851  02424878  02130319  02242962  02242963  02242964  66999995  66999996 |
