# Supplementary material for: A cohort study evaluating the association between concurrent mental disorders, mortality, morbidity, and continuous treatment retention for patients in opioid agonist treatment (OAT) across Ontario, Canada, using administrative health data
Source: Harm Reduct J. 2020 Jul 23;17:51. doi: 10.1186/s12954-020-00396-x (PMC7376938; doi:10.1186/s12954-020-00396-x)
Supplement: Supplementary file 2 — Additional file 2. Addiction Medicine Fee Codes [file 12954_2020_396_MOESM2_ESM.docx]

Appendix B

| Addiction Medicine Fee Codes | |
| --- | --- |
| **OAT monthly management codes** | K682 Opioid Agonist Maintenance Program monthly management fee - intensive, per month  K683 Opioid Agonist Maintenance Program monthly management fee - maintenance, per month  K684 Opioid Agonist Maintenance Program - team premium, per month, to K682 or K683 |
| **OAT visit/consultation codes** | A48 Consultation  A49 Repeat Consultation |
| **OAT point of care testing codes** | G040 Drugs of abuse screen, urine, must include testing for at least four drugs of abuse  G041 Target drug testing, urine, qualitative or quantitative  G042 Target drug testing, urine, qualitative or quantitative  G043 Drugs of abuse screen, urine, must include testing for at least four drugs of abuse |
