# Supplementary material for: A cohort study evaluating the association between concurrent mental disorders, mortality, morbidity, and continuous treatment retention for patients in opioid agonist treatment (OAT) across Ontario, Canada, using administrative health data
Source: Harm Reduct J. 2020 Jul 23;17:51. doi: 10.1186/s12954-020-00396-x (PMC7376938; doi:10.1186/s12954-020-00396-x)
Supplement: Supplementary file 3 — Additional file 3. Mental Health Service Fee Codes [file 12954_2020_396_MOESM3_ESM.docx]

Appendix C

| **Mental Health Service Fee Codes** | |  |
| --- | --- | --- |
| **Physician model of practice** | OHIP fee code | |
| **Primary Care Physician** | **K004** 2 or more family members in family therapy  **K005** Mental health individual care by general practitioner / family physician (GP/FP)  **K006** Individual care hypnotherapy  **K007** Psychotherapy individual care  **K010** Additional units for group therapy  **K012** 4 people group therapy  **K013** Counselling individual care by General Practitioner/Family Practitioner  **K019** 2 people group therapy  **K020** 3 people group therapy  **K024** 5 people group therapy  **K025** 6 to 12 people group therapy  **K680** extended assessment for substance abuse  **Q020** Premium for primary health care for patients with serious mental illness – bipolar disorder  **Q021** Premium for primary health care for patients with serious mental illness – schizophrenia | |
| **Psychiatry** | **A190** Special psychiatric consultation  **A193** Specific assessment  **A194** Partial assessment  **A195** Consultation  **A196** Repeat consultation  **A395** Limited consultation  **A695** Neurodevelopmental consultation  **A795** Geriatric psychiatric consultation  **A895** Consultation in association with special visit to a hospital in-patient, long-term care in-patient or emergency department patient  **K187** Acute post-discharge community psychiatric care, to K195, K196, K197 or K198  **K188** High risk community psychiatric care, to A190, A191, A192, A195, A197, A198, A695, A795, K195, K196, K197 or K198  **K189** Urgent community psychiatric follow-up, to A190, A195, A695 or A795  **K192** Individual hypnotherapy  **K195** Family psychotherapy - out-patients  **K197** Individual out-patient psychotherapy  **K203** 4 people group therapy  **K204** 5 people group therapy  **K205** 6 to 12 people group therapy  **K208** 2 people group therapy  **K209** 3 people group therapy  **K630** Psychiatric consultation extension  **K701** Mental health out-patient case conference | |
