# Supplementary material for: A cohort study evaluating the association between concurrent mental disorders, mortality, morbidity, and continuous treatment retention for patients in opioid agonist treatment (OAT) across Ontario, Canada, using administrative health data
Source: Harm Reduct J. 2020 Jul 23;17:51. doi: 10.1186/s12954-020-00396-x (PMC7376938; doi:10.1186/s12954-020-00396-x)
Supplement: Supplementary file 5 — Additional file 5. Study outcomes related to concurrent mental disorders and all-cause mortality for OAT patients (Regression Output) [file 12954_2020_396_MOESM5_ESM.docx]

Appendix E: Study outcomes related to concurrent mental disorders and all-cause mortality for OAT patients (Regression Output)

| **Odds Ratio Estimates** |  |  |  |
| --- | --- | --- | --- |
| **Effect** | **Point Estimate** | **95% Wald** | |
|  |  | **Confidence Limits** | |
| **Age Groups** |  |  | |
| 15-24 vs 65+ | 0.10 | 0.09 | 0.13 |
| 25-34 vs 65+ | 0.10 | 0.0.9 | 0.12 |
| 35-44 vs 65+ | 0.14 | 0.12 | 0.16 |
| 45-54 vs 65+ | 0.29 | 0.24 | 0.34 |
| 55-44 vs 65+ | 0.52 | 0.44 | 0.62 |
| 65+ |  |  |  |
| **Sex** |  |  |  |
| F vs M | 0.84 | 0.77 | 0.91 |
| **Location of Residence** |  |  |  |
| NR vs SU | 0.71 | 0.56 | 0.89 |
| NU vs SU | 0.92 | 0.83 | 1.06 |
| SR vs SU | 0.96 | 0.83 | 1.12 |
| **Income** |  |  |  |
| 1 vs 5 | 1.16 | 1.01 | 1.32 |
| 2 vs 5 | 1.08 | 0.93 | 1.24 |
| 3 vs 5 | 0.97 | 0.83 | 1.13 |
| 4 vs 5 | 0.92 | 0.79 | 1.09 |
| **HIV status** |  |  |  |
| Yes vs NO | 3.20 | 2.44 | 4.19 |
| **Deep Tissue Infection** |  |  |  |
| Yes vs NO | 2.24 | 1.93 | 2.60 |

Appendix E: Study outcomes related to concurrent mental disorders and ED visits for OAT patients (Regression Output)

| **Odds Ratio Estimates** |  |  |  |
| --- | --- | --- | --- |
| **Effect** | **Point Estimate** | **95% Wald** | |
|  |  | **Confidence Limits** | |
| **Age Groups** |  |  | |
| 15-24 vs 65+ | 1.82 | 1.60 | 2.08 |
| 25-34 vs 65+ | 1.71 | 1.51 | 1.95 |
| 35-44 vs 65+ | 1.59 | 1.40 | 1.82 |
| 45-54 vs 65+ | 1.34 | 1.72 | 1.53 |
| 55-44 vs 65+ | 1.08 | 0.94 | 1.25 |
| 65+ |  |  |  |
| **Sex** |  |  |  |
| F vs M | 1.55 | 1.49 | 1.61 |
| **Location of Residence** |  |  |  |
| NR vs SU | 3.47 | 3.14 | 3.83 |
| NU vs SU | 1.89 | 1.77 | 2.01 |
| SR vs SU | 2.15 | 2.01 | 2.30 |
| **Income** |  |  |  |
| 1 vs 5 | 1.58 | 1.49 | 1.68 |
| 2 vs 5 | 1.26 | 1.18 | 1.34 |
| 3 vs 5 | 1.13 | 1.06 | 1.21 |
| 4 vs 5 | 1.09 | 1.01 | 1.17 |
| **HIV status** |  |  |  |
| Yes vs NO | 2.75 | 2.14 | 3.53 |
| **Deep Tissue Infection** |  |  |  |
| Yes vs NO | 2.38 | 2.11 | 3.92 |

Appendix E: Study outcomes related to concurrent mental disorders and hospitalizations for OAT patients (Regression Output)

| **Odds Ratio Estimates** |  |  |  |
| --- | --- | --- | --- |
| **Effect** | **Point Estimate** | **95% Wald** | |
|  |  | **Confidence Limits** | |
| **Age Groups** |  |  | |
| 15-24 vs 65+ | 0.09 | 0.06 | 0.12 |
| 25-34 vs 65+ | 0.10 | 0.08 | 0.14 |
| 35-44 vs 65+ | 0.15 | 0.11 | 0.20 |
| 45-54 vs 65+ | 0.23 | 0.17 | 0.32 |
| 55-44 vs 65+ | 0.42 | 0.31 | 0.59 |
| 65+ |  |  |  |
| **Sex** |  |  |  |
| F vs M | 4.84 | 4.59 | 5.11 |
| **Location of Residence** |  |  |  |
| NR vs SU | 1.87 | 1.68 | 2.08 |
| NU vs SU | 1.48 | 1.37 | 1.60 |
| SR vs SU | 1.24 | 1.15 | 1.34 |
| **Income** |  |  |  |
| 1 vs 5 | 1.04 | 0.97 | 1.12 |
| 2 vs 5 | 0.99 | 0.92 | 1.07 |
| 3 vs 5 | 0.98 | 0.91 | 1.06 |
| 4 vs 5 | 0.96 | 0.88 | 1.04 |
| **HIV status** |  |  |  |
| Yes vs NO | 3.08 | 2.14 | 4.45 |
| **Deep Tissue Infection** |  |  |  |
| Yes vs NO | 3.68 | 3.00 | 4.51 |

Appendix E: Study outcomes related to the Concurrent Mental Disorders and One-year Treatment Retention for OAT Patients (Regression Output)

| **Odds Ratio Estimates** |  |  |  |
| --- | --- | --- | --- |
| **Effect** | **Point Estimate** | **95% Wald** | |
|  |  | **Confidence Limits** | |
| **Age Groups** |  |  | |
| 15-24 vs 65+ | 2.00 | 1.65 | 2.42 |
| 25-34 vs 65+ | 1.87 | 1.54 | 2.25 |
| 35-44 vs 65+ | 2.17 | 1.80 | 2.63 |
| 45-54 vs 65+ | 2.57 | 2.12 | 3.11 |
| 55-44 vs 65+ | 2.24 | 1.83 | 2.74 |
| 65+ |  |  |  |
| **Sex** |  |  |  |
| F vs M | 1.58 | 1.51 | 1.64 |
| **Location of Residence** |  |  |  |
| NR vs SU | 0.60 | 0.54 | 0.66 |
| NU vs SU | 1.01 | 0.84 | 1.08 |
| SR vs SU | 1.09 | 1.02 | 1.18 |
| **Income** |  |  |  |
| 1 vs 5 | 1.81 | 1.68 | 1.95 |
| 2 vs 5 | 1.41 | 1.30 | 1.53 |
| 3 vs 5 | 1.19 | 1.10 | 1.30 |
| 4 vs 5 | 1.05 | 0.96 | 1.15 |
| **HIV status** |  |  |  |
| Yes vs NO | 1.73 | 1.41 | 2.13 |
| **Deep Tissue Infection** |  |  |  |
| Yes vs NO | 0.93 | 0.82 | 1.05 |
